# A cost-effective polyethylene glycol-based method for the isolation of functional edible nanoparticles from ginger rhizomes

**DOI:** 10.1038/s41598-020-61358-8

**Published:** 2020-03-10

**Authors:** Sreeram Peringattu Kalarikkal, Durga Prasad, Ravi Kasiappan, Sachin R. Chaudhari, Gopinath M. Sundaram

**Affiliations:** 1 Academy of Scientific and Innovative Research (AcSIR), Ghaziabad, 201002 India; 2 Department of Biochemistry, CSIR-CFTRI, Mysuru, Karnataka India; 3 Department of Spice & Flavor Science, CSIR-CFTRI, Mysuru, Karnataka India

**Keywords:** Nanoparticles, Bioinspired materials

## Abstract

Edible nanoparticles (ENPs) are nano-sized vesicles derived from edible plants. These ENPs are loaded with plant derived microRNAs, protein, lipids and phytochemicals. Recently, ginger derived ENPs was shown to prevent inflammatory bowel diseases and colon cancer, *in vivo*, highlighting their therapeutic potential. Conventionally, differential centrifugation with an ultra-centrifugation step is employed to purify these ENPs which imposes limitation on the cost-effectiveness of their purification. Herein, we developed polyethylene glycol-6000 (PEG6000) based ginger ENP purification (PEG-ENPs) method, which eliminates the need for expensive ultracentrifugation. Using different PEG6000 concentrations, we could recover between 60% to 90% of ENPs compared to ultracentrifugation method. PEG-ENPs exhibit near identical size and zeta potential similar to ultra-ENPs. The biochemical composition of PEG-ENPs, such as proteins, lipids, small RNAs and bioactive content is comparable to that of ultra-ENPs. In addition, similar to ultra-ENPs, PEG-ENPs are efficiently taken up by the murine macrophages and protects cells from hydrogen peroxide induced oxidative stress. Since PEG has been approved as food additive, the PEG method described here will provide a cost-effective alternative to purify ENPs, which can be directly used as a dietary supplement in therapeutic formulations.

## Introduction

Edible nanoparticles (ENPs) are dietary plant-derived membrane vesicles with a size range between 100 to 500 nm. Plant derived ENPs are structurally similar to mammalian exosomes. Unlike exosomes, the exact intra/extra cellular origin of plant ENPs is currently unclear^1^. ENPs have gained considerable attention over the past decade in therapeutics, both as a delivery vehicle and as a bioavailable source of plant phytochemicals. ENPs were initially assumed to be produced by the apoplastic compartment of plants in response to pathogen infection^2^. Recent evidences indicate the presence of other bioactive elements, such as microRNAs, lipids and plant secondary metabolites^3–5^. ENPs are non-toxic compared to synthetically derived lipid nanovesicles, lack immunogenicity and show excellent stability through gastro intestinal digestion process *in vivo*^6^. The presence of intrinsic bioactives encapsulated within these nanovesicles immensely contributes to their excellent antioxidant, anti-inflammatory and anti-cancer activities *in vitro* and *in vivo*^5,7^. Hence, in the field of nanotechnology-based therapy, edible plant-derived ENPs possess tactical advantages due to their cheaper source material and eco-friendly isolation protocols employed, compared to chemically synthesized nanoparticles^8^. So far, ENPs have been isolated and characterized from several plant species such as ginger, grapefruit, grapes, lemon, tomato, broccoli, sunflower, orange, kiwi fruit, pear, soybean, coconut and hami melon, etc^1,3,8,9^. In case of ginger derived ENPs, the key bioactives of ginger plant, namely, 6-gingerol and 6-shogaol are present abundantly within these nanovesicles^10^. Oral delivery of ginger ENPs protects mice from inflammatory bowel disease and colitis associated colon cancer by suppressing inflammation and restoring intestinal homeostasis^7^. Moreover, ginger derived ENPs have also been exploited as a nanocarrier for targeted delivery of biological cargos, such as plasmid DNA, siRNAs, chemotherapeutic drugs and phytochemicals to unhealthy tissues^4,5,10–12^.

Exosomes are extracellular vesicles secreted by mammalian cells, which are derived from the endosomal compartment. Differential centrifugation is the most widely applied method for the separation of sub-cellular organelles, including exosomes^13^. This process involves sequential centrifugation of cell culture supernatants to remove unwanted cellular materials, followed by an ultracentrifugation step at ≥ 100,000 X g for selective enrichment of nanovesicles actively secreted by cells. Given the similarity between mammalian exosomes and plant ENPs, this method was later adopted for the purification of ENPs^8^. However, ENP purification using ultracentrifugation is an expensive method, primarily due to the cost associated with the ultra-centrifugation process (cost of instrument and consumables). Since plant ENPs are evolving as alternative therapeutics against several diseases, it is essential to have purification methods compatible with cheaper production costs. In case of exosomes, several alternative purification methods have been developed to replace the use of ultracentrifugation, such as ultra-filtration and polymer based precipitation methods^14–16^. In this line, polyethylene glycol 6000 (PEG) has been successfully used for the purification of mammalian exosomes as well as viruses. Mechanistically, PEG is a known crowding agent and has been proposed to form a mesh-like-net in which nanovesicles are trapped, and thereby precipitated^17^. PEG based purification method has never been attempted for edible plant ENPs. Since mammalian exosomes and ENPs are similar in structural characteristics, we were curious if PEG precipitation method could be adopted for plant ENP purification. In this report, we have developed a method to purify ginger ENPs via polymer-based precipitation using PEG6000. ENPs purified with PEG6000 (PEG-ENPs) are comparable to ENPs purified through ultra-centrifugation (ultra-ENPs), in terms of its biophysical/biochemical composition and biological characteristics. Our method could significantly reduce the cost associated with the ultracentrifugation-based purification of ENPs and, in general, can likely be adopted for the purification of ENPs from any dietary plant.

## Results

### Polyethylene glycol, Mn 6000Da (PEG6000) can be used for enrichment of ginger ENPs

**Figure 1:**
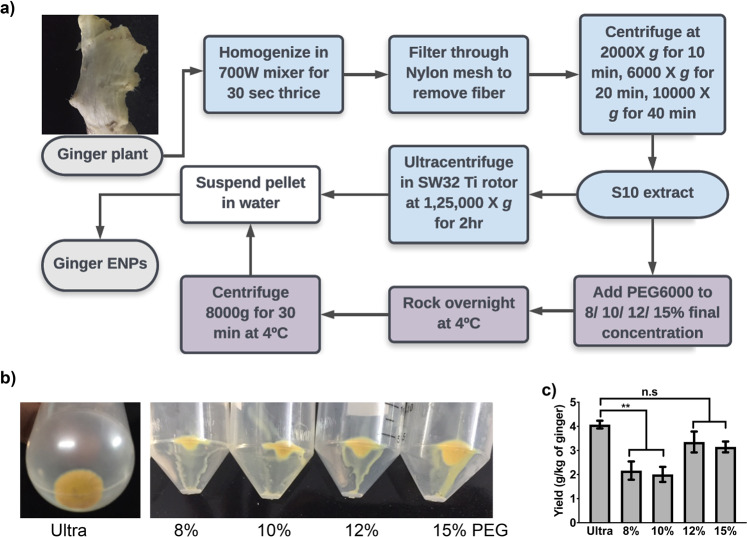
Purification of ginger derived ENPs using PEG6000. (**a**) Experimental flow chart depicting the purification of ginger ENPs using PEG6000 and ultra-centrifugation. (**b**) Photomicrographs of ENP pellets obtained in ultra-method (left panel) in comparison with increasing concentration of PEG6000. (**c**) The ENP pellets were lyophilized and weighed to calculate the total yield per kg of ginger rhizomes used. Increasing the PEG6000 concentration led to increased ENP yields. **P < 0.01. Results presented are an average of four independent experiments.

Poly ethylene glycol, Mn 6000 Da (PEG6000) based enrichment is a convenient and easy to scaleup procedure for purification of exosomes and viruses^15,18^. Bulk preparations of plant derived ENPs show broad size distributions ranging from 20 to 500 nm similar to exosomes and viruses^19^. Hence, we sought to examine, if PEG6000 can be used for selective enrichment of ENPs from ginger rhizomes. The experimental flow chart adopted in this study is depicted in Fig. 1a. Ginger rhizomes were first deskinned and subjected to homogenization (Fig. 1a). After filteration of excess fiber through a nylon mesh, the filtrate was subjected to differential centrifugation at indicated relative centrifugal forces (RCFmax) and time. At the end of the 10,000 X g centrifugation step, the soluble aqueous extract (S10 extract) was either subjected to conventional ultracentrifugation procedure (125000 X g for 2 hr.) or mixed with PEG6000 at different concentrations, incubated overnight at 4 °C and centrifuged at 8000 X g for 30 min. The concentration range of PEG (8%, 10%, 12% and 15%) was determined from earlier studies describing PEG based precipitation of exosomes^15,20–22^. As shown in Fig. 1b, we observed an increase in ENP pellet size with increasing concentration of PEG6000 (Fig. 1b). The pellets were lyophilized, and dry weight of the pellets were measured. In agreement with previous reports, the overall yield was approximately 4 g per kg of ginger in the case of ultra-ENPs, (Fig. 1c)^23^. The yields of ENPs varied between 2–3.8 g/kg of ginger when PEG6000 was used, indicating an efficiency of 60–90% for PEG method compared to ultra-method. In addition, a significant increase in ENP yield was obtained with 12% and 15% PEG compared to 8% and 10% (Fig. 1c). When the ability of other molecular weight PEGs (PEG4000 & PEG8000) to precipitate ENPs was compared with PEG6000 (final concentration 10%), ENP yield was higher with PEG6000 compared to PEG4000 and PEG8000 (Fig. S1a,b). We also investigated if centrifugation at lower RCFmax affects the yield of ENPs using 10% PEG6000. Precipitation of ENPs at <6000 g resulted in a significant decrease in ENP recovery (Fig. S1c,d).

It is worth noting here that ENP yield also increased with longer homogenization in both ultra and PEG method. However, this was avoided since longer duration led to significant heat generation during homogenization (data not shown). Hence, homogenization was restricted to 30 sec for 3 times with a resting period of 1 min. Even though ENPs were dialyzed to remove excess PEG6000, we investigated the presence of residual PEG content in the prepared ENPs. Ultra-ENP and PEG-ENP samples were resolved though 15% SDS-PAGE and PEG6000 was detected by Barium iodide staining as described earlier^22^. Residual PEG6000 was detected in prepared PEG-ENPs, despite the dialysis procedure (Fig. S2). Further quantification of residual PEG6000 with known standards revealed the presence of PEG6000 at a concentration between ~40 to 80 mg/gram (4% to 8%) in purified PEG-ENPs (Table S1). PEG6000 is a permissible food additive (E1521) with an acceptable daily intake of 10 mg/kg of body weight^24^. Hence, the presence of residual PEG6000 in ENPs, prepared with 10% PEG (560 mg/10 g) falls within the permissible limits of oral consumption. This is based on the assumed intake of 10 g of PEG-ENPs for an adult weighing 50–60 kg.

### Size and Zeta potential of PEG6000 precipitated ENPs vs ultra-method

**Figure 2:**
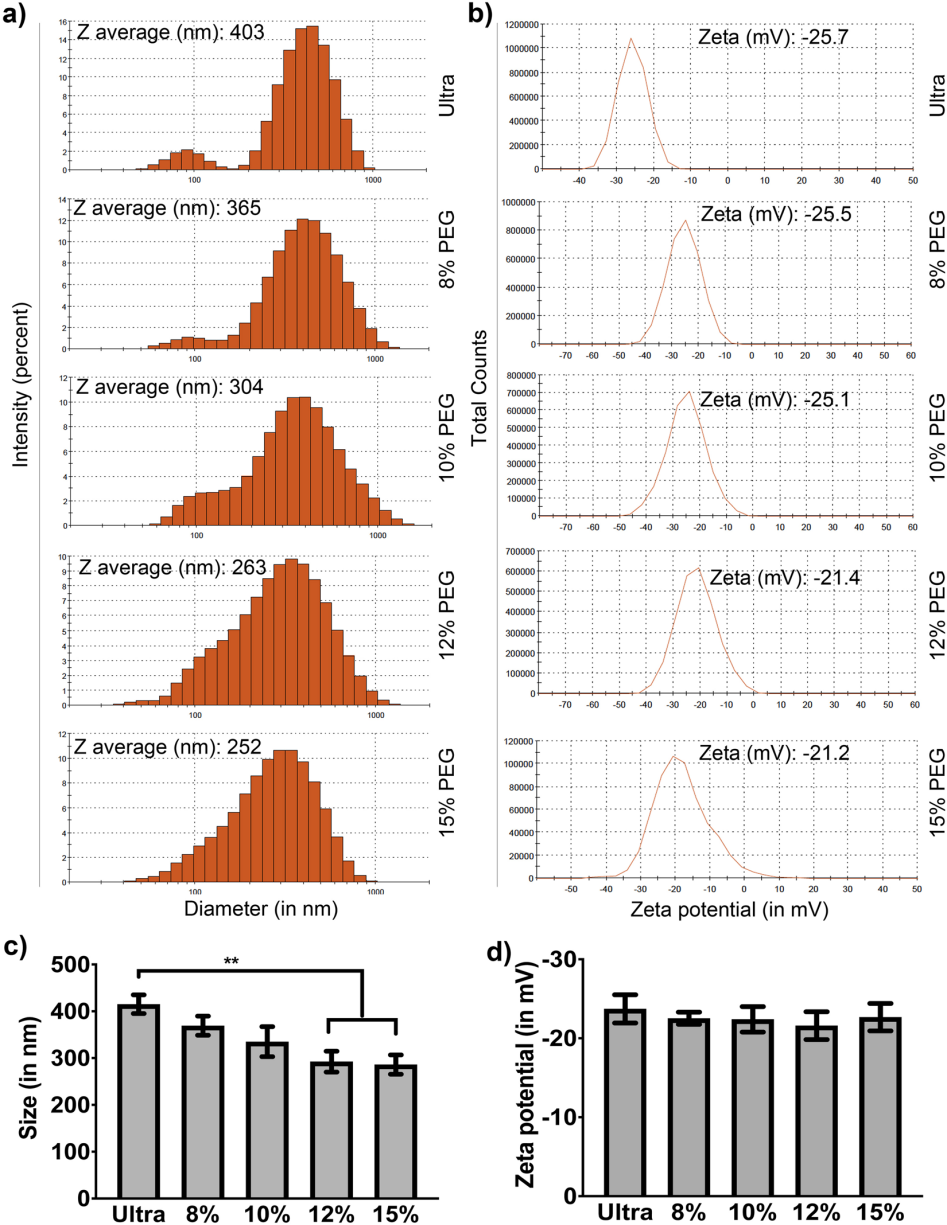
The size and zeta potential of PEG derived ENPs are comparable to ultra-ENPs. (**a**) & (**c**) Size distribution of purified ginger ENPs were determined using Malvern Zeta analyzer. PEG derived ENPs showed size distribution similar to ultra-ENPs, though at higher concentrations of PEG, significantly smaller sized ENPs were precipitated. **P < 0.01. Results presented are an average of four independent experiments. (**b**) & (**d**) No significant differences in zeta potential was observed between ultra-versus PEG-ENPs.

In order to characterize PEG-ENPs further, size heterogeneity and zeta potential were measured using Malvern Zeta sizer. Earlier studies have reported a heterogenous size population between ~100 to 1200 nm for ginger ENPs^5,7^. Further purification through sucrose density gradient was shown to yield two distinct populations of ~294 and 386 nm sized vesicles^5,23^. As shown in Fig. 2a, we observed a similar size distribution between 100 nm to 900 nm sized vesicles with a peak average size of ~400 nm for both ultra and PEG-ENPs (Fig. 2a). However, higher PEG concentration (12% and 15%) precipitated smaller sized ENPs compared to 8% and 10% PEG6000 (Fig. 2a,c). Zeta potential is a measure of relative aggregation tendency and stability of nanovesicle suspensions. Consistent with previous reports, ultra-purified ginger ENPs had a negative zeta potential value of ~ -25mV (Fig. 2b,d)^5^. There was no significant difference in Zeta potential between Ultra- ENPs versus PEG-ENPs (Fig. 2d). In addition, the size and zeta potential of ENPs isolated by PEG4000 or 8000 was similar to PEG6000 (Fig. S1e,g). Even though centrifugation of PEG-ENPs at lower rcfmax did not show a detectable change in zeta potential, centrifugation at 4000 g resulted in sedimentation of significantly larger sized ENPs (Fig. S1f,h).

### Intracellular delivery of PEG-ENPs and ultra-ENPs are equally efficient

**Figure 3:**
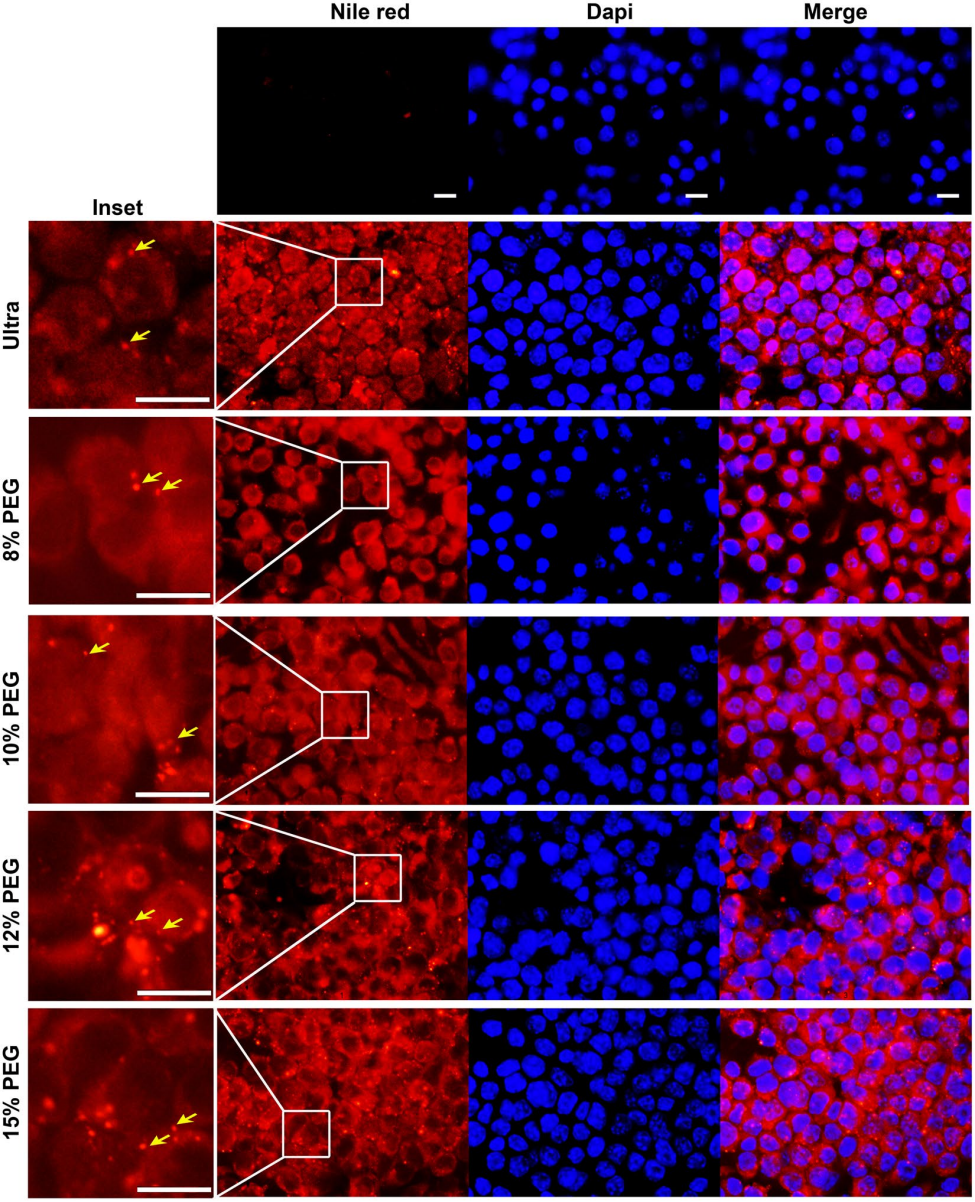
Intracellular uptake of PEG-ENPs is similar to ultra-ENPs in murine macrophages. RAW cells were either mock treated or treated with Nile red labeled ultra or PEG-ENPs (100 µg), for 24 hours. Cells were fixed and counterstained with the nuclear stain, DAPI. Top panel shows mock treated cells with no detectable fluorescence. Cytoplasmic Nile Red fluorescence was detected in all ENP treated cells indicating the intra-cellular uptake of both ultra and PEG-ENPs by murine macrophages (bottom panels). No significance difference in fluorescence intensity or the number of Nile red positive cells was observed between these two different treatments. Inset shows amplified regions depicted in the box to demonstrate the presence of Nile red labeled nano-sized ENPs. Scale bar-10 µm.

ENPs are emerging as an excellent delivery vehicle for biological macromolecules due to their ability to bind and fuse with various mammalian cell types^23,25^. ENPs also exhibit excellent bio availability and stability in gastro-intestinal environment *in vivo*^7^. Ginger ENPs have been shown to penetrate colonic epithelial cells and murine macrophages (RAW 264.7) *in vitro*^7,26^. This has led to their successful use as a delivery vehicle for siRNAs, *in vitro* and *in vivo*^26^. Hence, we compared the intra-cellular uptake dynamics of PEG-ENPs with ultra-ENPs. RAW macrophages were seeded on coverslips and treated with ultra-ENPs and PEG-ENPs (100 µg equivalent). ENPs were labeled with Nile red, a lipophilic fluorescent dye which enables us to track the intracellular fate of ENPs. Mock treated cells did not show a detectable fluorescence under the microscopic settings used for image acquisition (Fig. 3, top panel). After 24 hours post treatment, we observed detectable fluorescence in both ultra and PEG-ENP treated cells, indicating the efficient intra-cellular uptake of both type of ENPs (Fig. 3, lower panels). No significant difference in fluorescence intensity or the number of fluorescing cells per field were observed between ultra-ENPs and PEG-ENPs, irrespective of the PEG concentration chosen for precipitation (Fig. 3, lower panels). Moreover, a few ENPs, which has not gained entry into the cells but were likely bound to the surface of cells, were detectable at higher magnification in both cases (ultra or PEG) (Fig. 3, inset with arrows). The nano-sized nature of these unfused ultra/PEG-ENPs were approximately <400 nm, (in comparison with the 10 µm scale bar) in agreement with the peak size average of both ENPs obtained through Zetasizer analysis (Fig. 2c). To assess the intracellular uptake dynamics of both ultra and PEG-ENPs, we carried out fluorescence imaging at earlier time points, after addition of ultra or 10% PEG-ENPs. Intracellular fluorescence was detected as early as 10 min post addition of ENPs, in both cases (Fig. S3). The relative fluorescence intensity of cells increased linearly over time and saturated at 8 hr post addition for both ultra and PEG-ENPs. Thus, no significant difference in uptake dynamics or uptake efficiency was observed between PEG or ultra-ENPs.

### The small RNAs, proteins and lipids profiles of PEG-ENPs are similar to Ultra-ENPs

**Figure 4:**
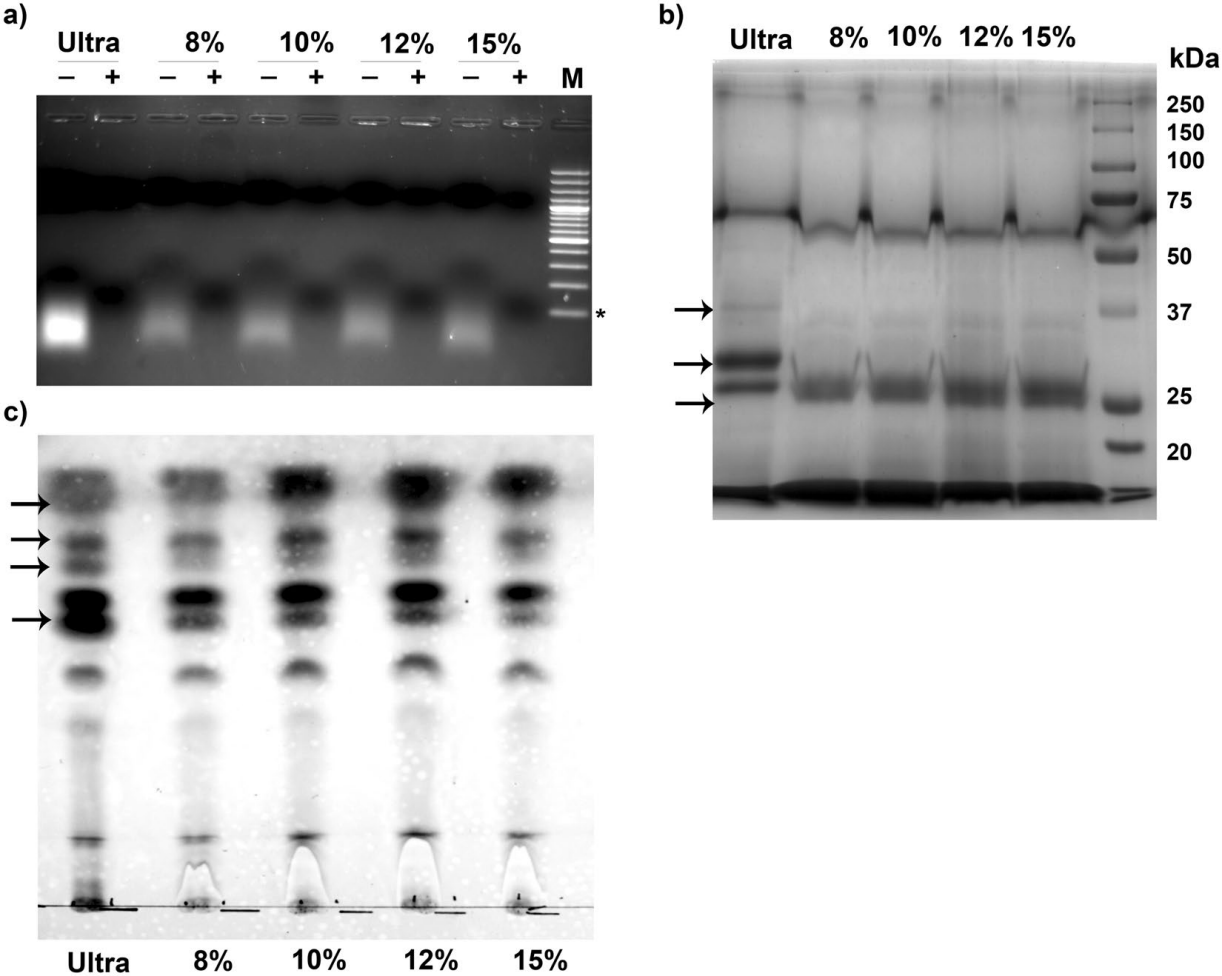
Comparison of small RNA population, proteins and lipids present within PEG-ENPs and ultra-ENPs. (**a**) Total RNA was isolated from ginger ENPs and equal amount of total RNA from each sample was treated with or without RNAse A. Samples were resolved through 1.5% agarose gel electrophoresis and visualized by ethidium bromide staining. The small RNA content of PEG-ENPs were identical to ultra-ENPs in size and sensitivity to RNAse A. M-100bp DNA ladder and * indicates 100 bp size. (**b**) Soluble proteins were extracted from equal amount of ginger ENPs, resolved through 10% SDS-PAGE and visualized by Coomassie brilliant blue staining. Bands with similar molecular weights were seen in both ultra-ENPs and PEG-ENPs. (**c**) Total lipids were extracted from ginger ultra/PEG-ENPs and resolved through silica gel F60 TLC plates. Representative picture for each analysis is shown from three independent experiments. Arrow marks in figures highlight proteins/lipids that show quantitative or qualitative difference between ultra-versus PEG-ENPs.

ENPs have also been shown to possess small RNAs, proteins and lipids derived from host plant. Using next generation sequencing, Xiao *et al*., (2018) demonstrated the presence of abundant microRNAs (miRNAs) in 11 different edible plant-derived ENPs and these miRNAs were predicted to target mammalian transcriptome^3^. Ginger ENPs have been shown to contain 125 different miRNA species, in which 124 miRNAs have putative human targets^7^. Notably, ginger ENP derived miR-7267–3p targets monooxygenase ycnE mRNA from Lactobacillus rhamnosus, a key component of gut microbiome, thereby modulating the gut immunity^27^. Hence, we investigated if PEG derived ENPs retains intact small RNA population. Total RNA was isolated by Trizol RNA extraction reagent with equal amount of PEG and ultra-ENPs and resolved through 1.5% Agarose gel electrophoresis. Both ultra and PEG-ENP derived RNAs were devoid of ribosomal RNAs (Fig. 4a). Presence of small RNA population below the 100 bp dsDNA band was observed in all the ENPs tested (Fig. 4a). Prior treatment of samples with RNAse A led to the disappearance of the band, confirming the RNA nature of the band observed (Fig. 4a). Even though, no significant difference in the quantity of small RNA population was observed between ultra and PEG-ENPs, the quality of RNA (as assessed by A260/280 ratio, was <1.7, as compared to 1.8–2.0 obtained for ultra-ENPs) was sub-optimal with 15% of PEG (data not shown). This could probably due to the ability of PEG to precipitate off-target proteins at higher concentration^15^.

Recently, Attenuated total reflection Fourier-transform Infrared Spectroscopy (ATR-FTIR) have been reported for simultaneous determination of protein and lipids from extracellular vesicles, without the need for extensive sample processing^28^. The distinctive absorption bands of proteins and lipid in the FTIR spectrum will also aid in the evaluation of Protein to Lipid (P/L) ratio. We explored the possibility of using ATR-FTIR technique for the characterization of lipids and proteins in ginger ENPs, which has never been attempted. Ginger ENPs has been shown to possess relatively lower protein content and lipid profile distinct from mammalian exosomes due to the lack of cholesterol in ginger rhizome^7^. After optimizing the acquisition conditions for ATR-FTIR on ginger ultra-ENPs (Table S2), we studied PEG-ENPs under identical conditions. The stack plot of ATR-FTIR spectrum for ultra-ENPs, 8% and 10% PEG-ENPs are shown in Fig. S4. Even though, the FTIR protein spectra for ultra and PEG-ENPs were similar (magnified inset showing bands between 1750 and 1500 cm-1), the absorbance bands (C-H stretch, observed between 3000–2800 cm-1) corresponding to lipids could not be used for quantification. This was mainly due to the presence of residual PEG6000 in the samples. PEG6000 also has -CH2- functionality which falls in the region of 3000–2800 cm^−1^ overlapping with lipid -CH2- functionality, highlighting the incompatibility of FTIR analysis for ENP samples containing PEG (Fig. S5). Therefore, the presence of proteins within these ENPs was confirmed by conventional protein profiling via SDS-PAGE. Earlier reports have extensively characterized proteins derived from PEG-purified mammalian exosomes by global proteomics approach^15,20,29^. PEG derived exosome proteome was shown to have 95–97% overlap with proteins known to be present in extracellular vesicles (source: exocarta, vesiculopedia). In agreement, Coomassie brilliant blue staining profile of ultra and PEG-ENPs showed the presence of proteins with identical molecular weight for the vast majority of proteins (Fig. 4b). However, we observed a minor fraction of proteins that are specifically co-sedimented either in ultra or PEG method (Fig. 4b, arrow marks). The exact nature of these proteins could not be predicted without in depth protein identification analysis via mass spectrometry. We also compared the total lipid profile of ultra-ENPs with PEG-ENPs. Total lipids were extracted from equal amount of ENPs (10 mg) and resolved through thin layer chromatography as described in methods. The lipid composition of PEG-ENPs were mostly similar to ultra-ENP derived lipids irrespective of the PEG concentration used (Fig. 4c). A minor fraction of lipids showed quantitative difference between ultra and PEG-ENPs suggesting the selective preference of PEG for certain species of lipids compared to ultra-centrifugation (Fig. 4c, arrow marks).

### Both ultra and PEG-ENPs contain functional ginger bioactives

**Figure 5:**
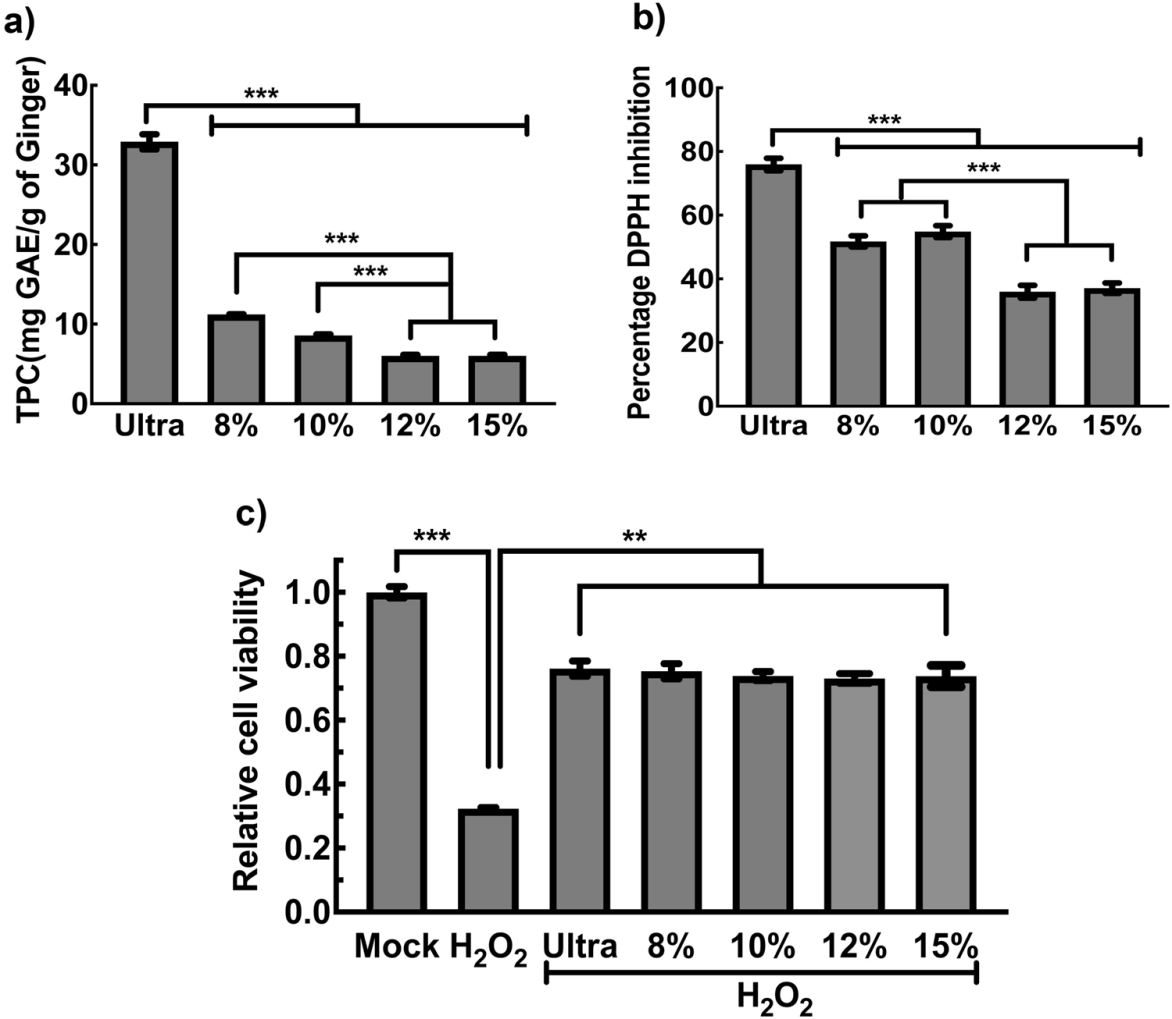
Total polyphenols are present in PEG-ENPs and exhibit antioxidant activity similar to ultra-ENPs, *in vitro*. (**a**) Total polyphenolics (TPC) were extracted from equal amount of ginger ENPs and assayed by Folin-Ciocalteu reagent as described in methods. Use of higher PEG6000 concentrations led to decreased enrichment of TPCs in ENPs. (**b**) Antioxidant activity of ultra and PEG-ENPs were measured using DPPH assay as described in methods. The antioxidant activity of TPCs encapsulated within ENPs were significantly reduced when 12% and 15% PEG were used, compared to 8%/10% PEG derived ENPs. (**c**) Antioxidant functionality of ENP associated bioactives on rescuing H_2_O_2_ induced cell death on RAW macrophages. Cell viability of RAW macrophages treated with H_2_O_2_ alone or in the presence of ENPs were measured by MTT assay (n = 3). ***P < 0.001, **P < 0.01.

According to recent reports, ENPs are an excellent source of plant bioactives in bioavailable form. In particular, polyphenolics such as 6-gingerol and 6-shogaol has been isolated in high quantities from ginger ENPs purified by conventional ultracentrifugation^5,7,30^. These ginger ENP derived bioactives possesses excellent antioxidant properties both *in vitro* and *in vivo*. Hence, we further ascertained the presence of polyphenolics in PEG-ENPs using biochemical methods. Total polyphenolic content (TPC) was measured from equal amount of ultra or PEG-ENPs using Folin-Ciocalteu method as described earlier^31^. As shown in Fig. 5a, compared to ultra-ENPs, 8% PEG-ENPs contained about 30% of TPCs and the total TPC content significantly decreased with increasing concentration of PEG used (especially at 12% and 15%). The decrease in TPC content with higher PEG concentrations could be either due to the suboptimal recovery of TPCs from samples containing higher amount of residual PEG or inhibition of FC assay by residual PEG present in the sample. Since total polyphenolic content are positively correlated with antioxidant capacity, we also measured the DPPH-free radical scavenging activity in ultra and PEG-ENPs as per Alhakmani *et al*.^31^. As expected, all the PEG-ENPs showed comparable inhibition of DPPH free radical activity with ultra-ENPs. Consistent with Fig. 5a, higher PEG concentration (12% and 15%) led to a significant decrease in free radical scavenging activity, likely due to the decreased TPC content (Fig. 5b).To confirm the functionality of the bioactives present within PEG-ENPs, we further investigated, if ENP derived bioactives could protect cells against oxidative stress induced cell death^32^. RAW macrophages were either treated with hydrogen peroxide (H_2_O_2_) alone to induce oxidative stress or co-treated with equal concentrations of ultra or PEG-ENPs. Treatment of RAW macrophages with H_2_O_2_ (200 µM) led to significant decrease in cell viability. Co-treatment with ultra-ENPs was able to rescue cells from oxidative stress induced cell death (Fig. 5c). We noticed that both ultra-ENPs and PEG derived ENPs rescued H_2_O_2_ induced cell death in both serum free and serum containing culture conditions (Fig. 5c & Fig. S6). Collectively, our findings support the conclusion that the PEG derived ENPs are functionally similar to ultra-ENPs.

## Discussion

The presence of nano-sized vesicles in edible plants was observed five decades ago and were thought to be microscopic artifacts^33,34^. It is evident from recent literature that not only these plant-derived ENPs are rich in bioactive compounds but ENPs are also an excellent delivery vehicle for biological cargos, such as DNA, siRNAs, proteins and chemotherapeutic drugs^8^. Although, ENPs share structural similarities to extra-cellular vesicles, the authentic extra- cellular nature of these ENPs has not been convincingly demonstrated. The probable reason for this gap is due to the fact that unlike mammalian exosomes which are harvested from conditioned medium without host cell lysis, ENP isolation requires mechanical disruption of plant material through extensive homogenization. Hence, it is an unrealistic goal to achieve the separation of ENPs that are purely derived from extracellular space. Though, purification of extracellular ENPs from sunflower seeds has been attempted via selective extraction of apoplastic fluid by vacuum infiltration procedure, adaptation of this method for ginger ENP purification is not possible due to high water content of ginger rhizomes (79%)^35^. Moreover, the key purpose of purifying ENPs from medicinally important plants, such as ginger rhizome, is to obtain ginger bioactives in a bio available form intended for therapeutic use. Hence, obtaining high ENP yield at low cost is of prime importance, a factor that could be traded off with low purity due to the edible nature of ginger ENPs.

Differential ultracentrifugation is the current gold-standard method for plant ENP purification, which poses a major obstacle in cost-effective purification of these ENPs for therapeutic use. In this study, we have described a convenient PEG6000 based precipitation method for ENP purification, with comparable efficacy to ultra-centrifugation method. The cost-effective nature of PEG6000 is primarily attributed to the elimination of the ultracentrifugation step while adopting this method. Table S3 summarizes the key factors that contribute significantly to the cost-effectiveness of PEG method compared to ultracentrifugation. This includes, the cost of instrument and consumables involved in ultracentrifugation, availability of rotors that can accommodate larger volume of samples, the technical expertise required to operate the instrument and the availability of ultra-centrifuge in individual research laboratories.

In the method described here, the percentage of PEG6000 appears to be a key factor in determining the quality and quantity of ginger ENPs obtained. Even though, all PEG6000 concentrations were able to purify ENPs containing bioactive compounds, use of higher PEG concentrations (12% and 15%) resulted in a significant inhibitory effect on the total polyphenolics content, antioxidant capacity and the quality of small RNA population. Moreover, PEG-ENPs have comparably less TPCs (30%) compared to ultra-ENPs. Moreover, PEG method also yields lesser amount of ginger ENPs compared to ultracentrifugation irrespective of the concentration of PEG6000 used.

This in contrast to earlier reports which indicate that PEG method is superior to ultracentrifugation in delivering higher yield of exosomes or viruses^15,20,22^. One likely reason for this discrepancy could be due to the intrinsic differences between the lipid composition of plant-derived ENPs compared to mammalian exosomes. Unlike mammalian exosomes, which are rich in phosphatidylcholine, ginger ENPs are rich in phosphatidic acid (42%) and glycerol lipids such as mono/digalatosyl diacylglycerol (MGDG/DGDG). In addition, cholesterol forms an integral part of mammalian exosomes and plays a key role in stabilizing lipid bilayer whereas plant derived ENPs are free from cholesterol^8^. Hence, efficient precipitation of plant ENPs may require further optimization, either by the addition of co-precipitant/s or by changing the conditions of precipitation. In this line, exosome/virus purification methods have been exhaustively studied and several variations of the precipitation method have been shown to increase the recovery. These include, usage of PEG polymers with different molecular weight, addition of sodium chloride (75 mM to 1 M), protamine sulphate, dextran sulphate and/or under acidic pH conditions^21,22,36–38^. We have observed no significant difference in the yield of ginger ENPs when PEG4000 or 8000 is used instead of PEG6000. Whether, the other parameters mentioned above, enhances the recovery of plant ENPs when combined along with PEG6000, is yet to be determined. Taken together, we conclude that the usage of 10% PEG6000 will be an optimal high recovery but low specificity method for plant-derived ENP purification without compromising on the quality. The presence of plant-derived bioactives within these ENPs in a bioavailable form, has led to several investigators testing their therapeutic effect against inflammatory and cancerous diseases^1,5,7,9,27,39,40^. Since PEG6000 is also used as a food additive with excellent safety profiles^24^, we speculate that the PEG method described here, can likely be adopted for preparative scale purification of therapeutically valuable ENPs, for their incorporation as a food additive targeting major diseases.

## Materials and methods

### Cell culture

The murine macrophage cell line, Raw 264.7 was obtained from National Center for Cell Sciences, Pune and cultured in Dulbecco’s modified Eagle’s medium (Sigma Aldrich) supplemented with 10% fetal bovine serum (Sigma Aldrich) and antibiotics (Penicillin and Steptomycin), in 5% CO2 environment at 37 °C.

### Isolation of ginger ENPs by differential ultra-centrifugation and PEG method

ENPs from fresh ginger rhizomes were isolated as per previously published protocols with minor modifications^5^. About 250 g of fresh ginger rhizomes were procured from local market and washed thoroughly with water. After peeling, the rhizomes were ground using a mixer grinder (750-watt power, maximum rpm 18,500 under no load conditions and 11,000 rpm with load) at medium speed for 3 mins with 30 sec on/off cycles. Excess fiber was removed from the juice by filtering through a nylon mesh (pore size 125 µm). The fresh juice thus obtained was centrifuged at 2000g for 10 min, 6000 g for 20 min and 10,000 g for 45 min to remove large fibers/cells, large particles/cell debris and microparticles, respectively^19,39^. The supernatant obtained after the 10,000 g step is referred as S10 extract. The yield of S10 extract varied between 70–80% of the initial ginger weight (e.g. 180 to 200 ml of S10 extract per 250 g of ginger) due to the removal of peel and insoluble debris. To purify ginger ENPs using conventional method, the S10 extract was subjected to ultracentrifugation in Beckman SW32.1 Ti swinging bucket rotor at 1,25,000 g for 2 hours at 4 °C. For isolation of ENPs using PEG, the S10 supernatant was mixed with PEG6000/4000/8000 (Sigma Aldrich) to reach a final concentration of 8%, 10%, 12% and 15% (weight by volume) and were incubated overnight at 4 °C with gentle rocking. After centrifugation at 8000 g for 30 min at 4 °C in a fixed angle rotor (Eppendorf), the excess PEG/liquid was removed by inverting the tube on a piece of tissue paper for 5 min. Both ultra and PEG-ENP pellets were suspended in sterile water and dialyzed overnight against sterile milli Q grade water using Dialysis membrane (Himedia) with a 10 kDa pore size. ENPs were lyophilized, weighed and resuspended in milli Q grade water to achieve a final concentration of 0.5 mg/µl of ENPs. The methodology developed here has been submitted to the EV-TRACK knowledgebase and this can be accessed with the EV-TRACK ID: EV190070^41^.

### PEG6000 staining by barium iodide method

The residual PEG6000 present in PEG-ENPs were measured by a modified protocol adopted from earlier reports^22,42^. Briefly, 200 µg of ultra or PEG-ENP samples or known concentrations of pure PEG6000 was boiled in 2X Lamelli buffer and resolved through 15% SDS-PAGE. The gel was washed twice with distilled water and immersed in 5% Barium chloride solution for 20 mins. After a quick rinse in distilled water, PEG6000 was visualized by staining with 0.1 M iodine solution for 5 min. Excess stain was removed by rinsing the gel in distilled water several times. Images were acquired using Syngene G:Box Chemi XT4 gel documentation system fitted with an epi LED white light. Band intensities were quantified using Image J software and the relative concentration of PEG6000 in PEG-ENPs was derived from the band intensities of known concentrations of PEG6000 loaded on the same gel.

### Intracellular uptake of ginger ENPs

In order to track the cellular fate of ginger vesicles, ENPs were first labeled with the lipophilic dye, Nile Red (Sigma Aldrich). For labeling ENPs, Nile red was added to the S10 extract before the ultra-centrifugation or PEG precipitation step (final concentration, 1 µM) so that Nile Red bound ENPs are selectively precipitated while unbound dye remains in the supernatant. For intra-cellular uptake experiment, RAW macrophages (200,000 cells) were seeded into 24 well TC plate containing glass coverslips. After 24hrs of culture, cells were treated with ENPs equivalent to 100μg, followed by incubation for different time points as indicated in figure legends. Cells were washed twice with PBS and fixed with 4% paraformaldehyde for 20 min. Cells were counterstained with DAPI (Sigma Aldrich) at a final concentration of 100 ng/ml and mounted onto a glass slide using Floursave fluorescent mounting media (Sigma Aldrich). Images were acquired using a fluorescence inverted microscope (Olympus IX73) under DAPI and TRITC channel (for Nile red) under 10X or 63X magnifications. Images were processed using ImageJ software.

### *In vitro* antioxidant activity assay

The antioxidant activity of ginger ENPs was evaluated using the hydrogen peroxide (H_2_O_2_) induced cell death on raw macrophages as described earlier^32^. Briefly, RAW macrophages were seeded onto 96 well plates at a density of 25000 cells per well. 24 hours post seeding, cells were washed once with PBS and treated with either H_2_O_2_ alone (200 µM) or co-treated with ginger ENPs equivalent to 10 µg, per well. After incubation for 3 hours, cells were washed and relative cell viability was measured using MTT (3-(4,5-dimethylthiazol-2-yl)-w,5-diphenyltetrazolium bromide) reagent as per standard protocols^43^.

### Surface charge and particle size analysis of ginger ENPs

The particle size and zeta potential were measured using Malvern zeta sizer nano ZS (Malvern Instruments, Malvern, UK) as described earlier^23^. ENPs were diluted 100-fold in milli Q water and triplicate measurement were made at room temperature for both hydrodynamic radius and zeta potential. Size and zeta potential measurements reported are the mean ± standard deviation from three to four different batches of ginger ENPs.

### Total polyphenolic content (TPC) estimation of ginger ENPs

Total polyphenolics from ginger ENPs were purified by methanol extraction. Briefly, 20 µl of ENPs were mixed with 100 µl of absolute methanol, vortexed and incubated at room temperature for 10 min. After centrifugation at 10,000 × g for 5 minutes, supernatant fraction was utilized for TPC estimation using a modified protocol described by Alhakmani *et al*.^26^. In brief, the supernatant fraction was mixed with 400 µl of Folin-Ciocalteu reagent (HiMedia laboratories) (diluted tenfold with water) and vortexed. After the addition of 800 µl of 7.5% sodium carbonate, samples were incubated at room temperature for 30 mins. Samples were transferred to 96 well colorimetric plates and the blue color developed was measured using an ELISA plate reader at 765 nm wavelength. Gallic acid was used to generate standard curve and TPC values are represented as gallic acid equivalents per gram of ginger.

### 1,1-diphenyl-2-picrylhydrazyl (DPPH) assay for antioxidant activity of ginger ENPs

The free radical scavenging activity of ENPs was evaluated using a protocol adopted from Shimamura *et al*.^44^. Briefly, 7.89 mg of DPPH reagent was dissolved in 100 ml of methanol to achieve a final concentration of 0.2 mM. Solution was kept in dark for 2 h for stabilization of colorimetric absorbance. Phytochemicals were purified from ENPs by extraction with Methanol as mentioned earlier. 100μl of methanol extract was mixed with 900μl of DPPH reagent and incubated at room temperature for 30 min. Absorbance was measured at 517 nm using an ELISA plate reader (TECAN). DPPH reagent alone served as a control. All the absorbance values were subtracted from background reading obtained with methanol alone. The DPPH antioxidant activity was calculated using the following formula, where (A) control is the absorbance of DPPH reagent alone and (A) sample is the absorbance of DPPH reagent + ENPs.

$$Radical\,scavenging( \% )=[\frac{({\rm{A}})\,{\rm{control}}-(A)sample}{({\rm{A}}){\rm{control}}}]\times 100$$

### Total RNA extraction and agarose gel electrophoresis of ginger ENP derived RNA

For isolation of total RNA from ginger ENPs, 500 µl of TRI reagent (Sigma) was mixed with 100 µl of ENPs. After the addition of 200 µl of chloroform, samples were vortexed vigorously and subjected to centrifugation at room temperature at 10,000 X g for 10 min. The aqueous phase containing total RNA was precipitated using equal volume of isopropanol. The RNA pellet obtained was washed twice with 75% ethanol and the pellet was suspended in 30 µl of nuclease free water. Total RNA was quantified using NanoDrop spectrophotometer. To authenticate the validity of the total RNA isolated, 1 µg of RNA was incubated with or without 0.5 µg of RNAse A and the samples were resolved through 1.5% agarose gel electrophoresis. Images were acquired using Syngene G:Box Chemi XT4 gel documentation system fitted with a UV transilluminator.

### SDS-PAGE analysis of ginger ENPs

To extract the proteins from ginger ENPs, samples were treated with buffer containing 50 mM Tris pH, 7.4, 500 mM NaCl, 1% Triton X 100, 1% NP40 and protease inhibitor cocktail at room temperature for 20 min. Samples were centrifuged at 12000 X g for 10 min to remove insoluble debris. Total protein concentration of prepared ENPs were determined using Bradford protein assay kit (BioRad) as per manufacturers protocol. Known concentrations of bovine serum albumin was used to generate the protein standard curve. Soluble proteins were treated with 2X Lamelli sample buffer, boiled at 95 °C for 5 min and were resolved through 10% SDS-PAGE. Gel was fixed and proteins were stained in a solution containing 10% acetic acid, 40% methanol and 0.25% Coomassie brilliant blue R250.

### TLC analysis of total lipids

Total lipids extraction and TLC analysis were performed according to the protocol described by Mu *et al*.^23^. In brief, 10 mg of ENPs were mixed with equal volume of chloroform and methanol and centrifuged at 2000 rpm for 5 min at room temperature. The organic phase obtained was dried under a stream of nitrogen and suspended in chloroform. The extracted lipids were resolved through silica gel 60 F254 TLC plates (Merck) using chloroform/methanol/acetic acid (95:4.5:0.5, by volume) as a solvent system. Plates were dried at room temperature and sprayed with a solution containing 10% copper sulfate and 8% phosphoric acid. Lipid bands were visualized by charring the plates at 120 °C for 10 min.

### Spectral evaluation of ENPs by FTIR

ATR-FTIR analysis was carried out using Platinum ATR mounted Bruker Tensor-II series FTIR spectrometer. For protein to lipid ratio calculation, we adopted the methodology described earlier by Mihaly *et al*.^28^ 10 µl of sample was mounted on the diamond ATR crystal and waited for ~45 minutes to form the thin dry film. The measurements were performed in ambient condition, immediately after thin film formation. The absorbance of the samples and background were measured using 128 scans co-added to each measurement. The absorption spectral range was collected between 4000 cm^−1^ and 450 cm^−1^, with a spectral resolution of 4 cm^−1^. For all spectral evaluations, Opus spectroscopy software was used. Background subtraction, baseline correction and spectrum smoothening was performed as described earlier^28^. Proteins were assessed by fitting of Lorentzian bands at 1635 cm^−1^ for amide-I and 1550 cm^−1^ for amide-II. Lipid contents were assessed by calculating the total integral intensity of stretching vibration from 3020 cm^−1^ to 2700 cm^−1^. For PEG-ENPs, the protein/lipid spectrum was subtracted with spectrum obtained with 10% PEG alone.

### Statistical methods

The data described here are the average results of three or more independent experiments with minimum triplicates measurements performed in each assay. Data are plotted using GraphPad Prism software. Statistical testing between samples was conducted with ANOVA algorithm in GraphPad, with Turkey’s multiple testing correction.

### Supplementary information

Supplementary information.

## Data Availability

Complete data of the manuscript is available with the corresponding author.
