# Author Correction: A cost-effective polyethylene glycol-based method for the isolation of functional edible nanoparticles from ginger rhizomes

**DOI:** 10.1038/s41598-024-66717-3

**Published:** 2024-07-12

**Authors:** Sreeram Peringattu Kalarikkal, Durga Prasad, Ravi Kasiappan, Sachin R. Chaudhari, Gopinath M. Sundaram

**Affiliations:** 1 Academy of Scientific and Innovative Research (AcSIR), Ghaziabad, 201002 India; 2 Department of Biochemistry, CSIR-CFTRI, Mysuru, Karnataka India; 3 Department of Spice & Flavor Science, CSIR-CFTRI, Mysuru, Karnataka India

Correction to: *Scientific Reports* 10.1038/s41598-020-61358-8, published online 10 March 2020

The original version of this Article contained an error in Affiliation 1, which was incorrectly given as ‘Academy of Scientific and Innovative Research (AcSIR), CSIR-CFTRI Campus, Mysuru, Karnataka, India’. The correct affiliation is listed below:

Academy of Scientific and Innovative Research (AcSIR), Ghaziabad-201002, India.

The original Article has been corrected.

