# Supplementary material for: A cost-effective polyethylene glycol-based method for the isolation of functional edible nanoparticles from ginger rhizomes
Source: Sci Rep. 2020 Mar 10;10:4456. doi: 10.1038/s41598-020-61358-8 (PMC7064537; doi:10.1038/s41598-020-61358-8)
Supplement: Supplementary file 1 — Supplementary information. [file 41598_2020_61358_MOESM1_ESM.pdf]

A cost-effective polyethylene glycol-based method for the isolation of functional edible nanoparticles from ginger rhizomes

Sreeram Peringattu Kalarikal[1&2], Durga Prasad[3], Ravi Kasiappan[2], Sachin R. Chaudhari[3], Gopinath M Sundaram[1,2*]

[1]Academy of Scientific and Innovative Research (AcSIR), CSIR-CFTRI Campus, Mysuru, Karnataka, India.

[2]Department of Biochemistry, CSIR-CFTRI, Mysuru, Karnataka, India.

[3]Department of Spice & Flavor Science, CSIR-CFTRI, Mysuru, Karnataka, India.

Fig. S1:

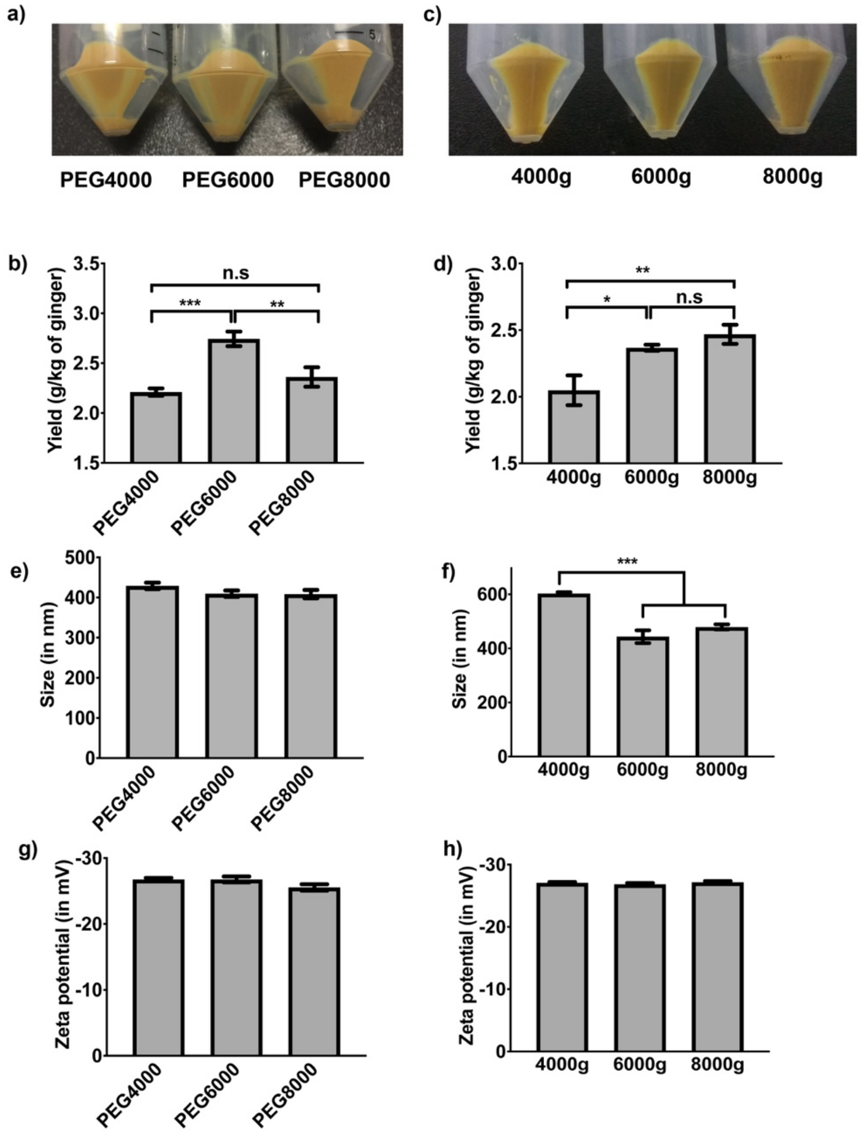

Fig. S1: Effect of PEG molecular weight and different rcf_{max} values on ginger ENP characteristics. Photomicrographs of ENP pellets obtained with PEG4000, 6000 or 8000 (a) or ENP pellets obtained by subjecting 10% PEG6000 samples to different rcf_{max} values of 4000g, 6000g and 8000g (c). The ENP pellets were lyophilized and weighed to calculate the total yield per kg of ginger rhizomes under similar conditions (b and d). The mean size of ginger ENPs precipitated with different molecular weight PEGs or precipitated under different rcf_{max} conditions with 10% PEG6000 samples (e & f). Zeta potential values of

ginger ENPs precipitated with different molecular weight PEG or centrifuged under different rcf_{max} conditions (g & h). Values are average of three independent batches of ginger rhizomes purified under given conditions. ***P<0.001, **P<0.01, *P<0.05, n.s: non-significant.

Fig. S2:

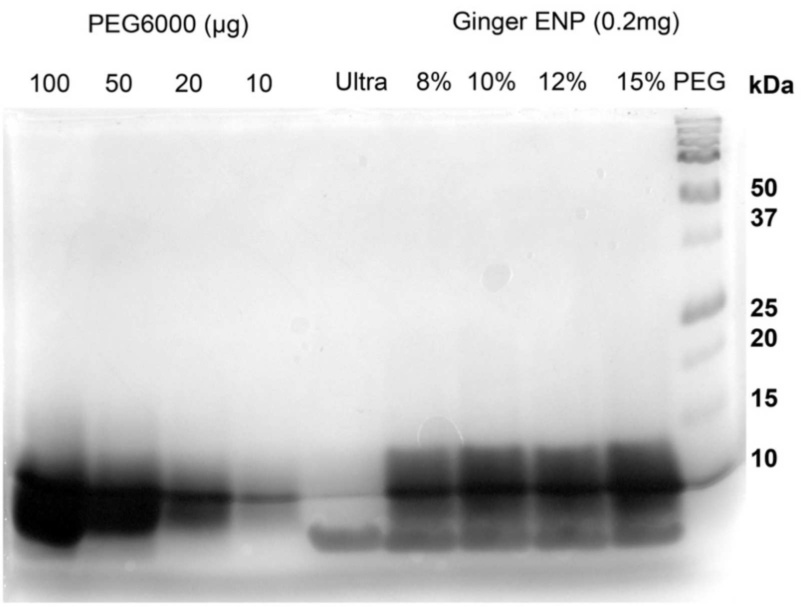

Fig. S2: Analysis of residual PEG6000 in PEG purified ENP samples. 200µg of ENP samples were resolved through 15% SDS-PAGE along with PEG6000 standards at indicated amounts. PEG6000 was visualized using Barium iodide staining procedure as described in method section. Band intensities were quantified using Image J software after background subtraction. The relative percentage of PEG in PEG-ENPs samples (in Table S1) was calculated using the standard curve obtained with standard PEG6000 band intensities.

Fig S3:

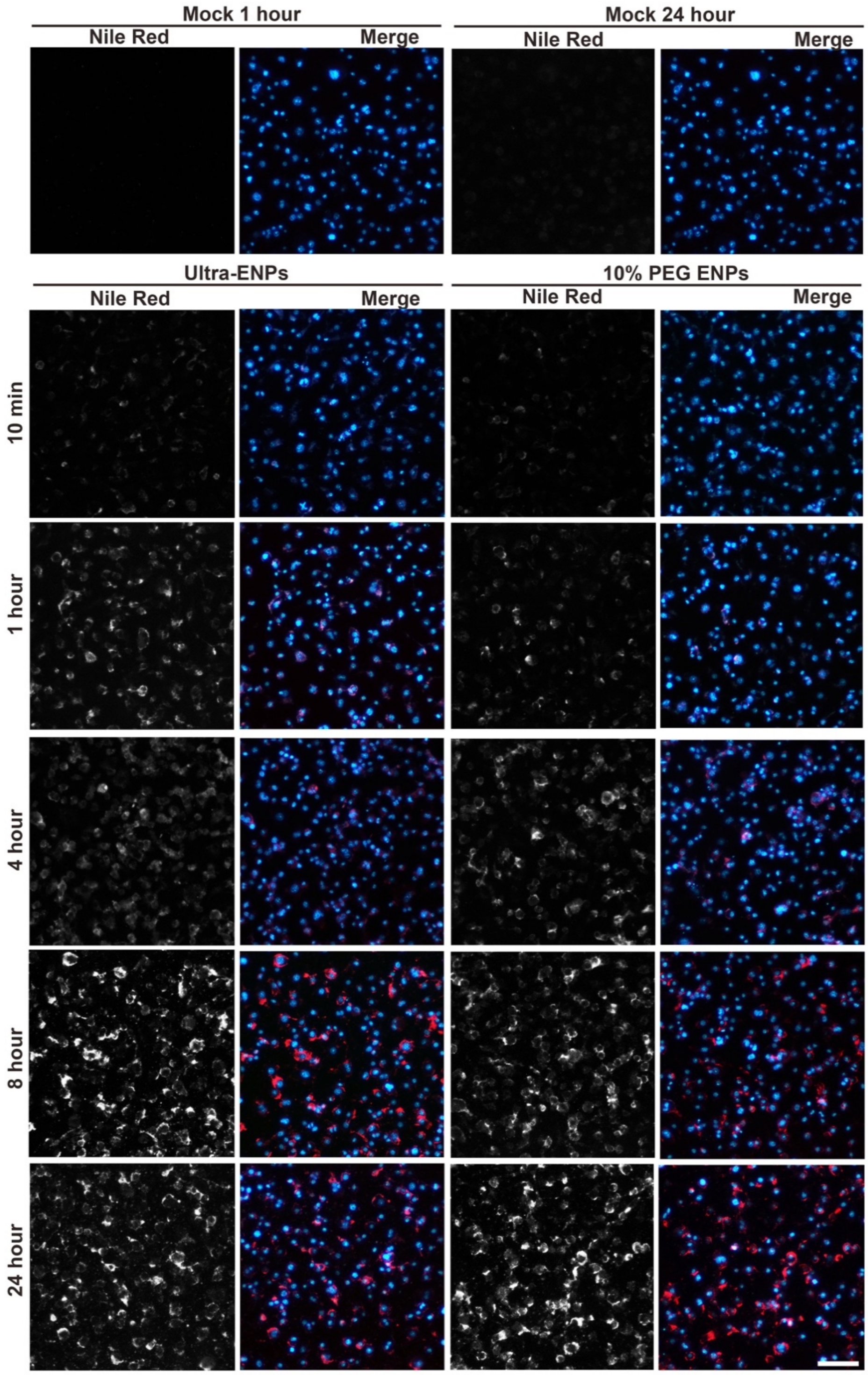

Fig. S3: Comparison of intracellular uptake of 10% PEG-ENPs with ultra ENPs at different time points post addition. Murine macrophages were either mock treated or treated with Nile

red labeled ultra or PEG-ENPs (100µg), for indicated time points. Cells were fixed and counterstained with the nuclear stain, DAPI. Images were acquired under same exposure settings under 10X magnification. Fluorescence intensity representing only the red channel (Nile red) is shown in grey scale while merged color images are shown for DAPI and Nile red. Scale bar-50µm. Images are representative of three independent experiment. In each experiment, images from five random fields were acquired.

Fig S4:

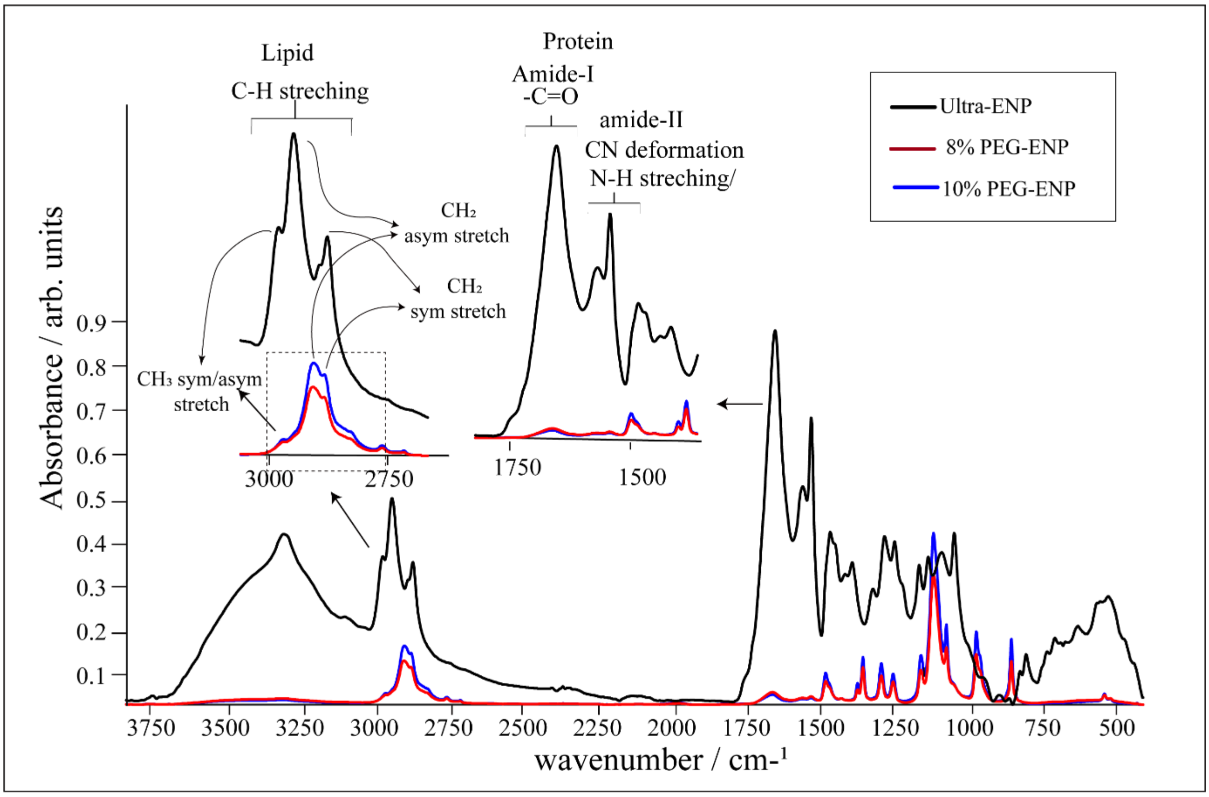

Fig. S4: ATR-FTIR Absorbance spectrum of Ultra-ENP, 8% PEG 10% PEG-ENP sample. The selected region bands of interest of CH_2/CH_3 and =CO are expanded.

Fig S5:

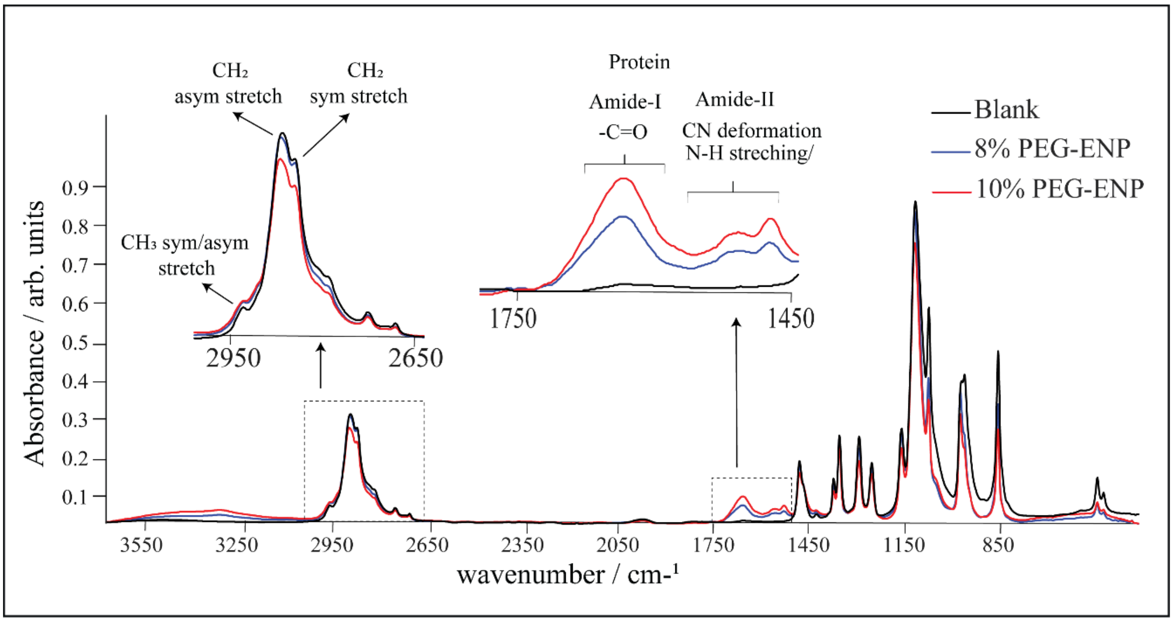

Fig S5: ATR-FTIR Absorbance spectrum of blank containing 10% PEG6000 as a blank (black), 8% PEG-ENP sample (blue) and 10% PEG-ENP (red) sample. The selected region of CH_2/CH_3 representing CH-stretch contributed from lipids or PEG and =CO amide- I functional group likely representing proteins are expanded.

Fig S6.

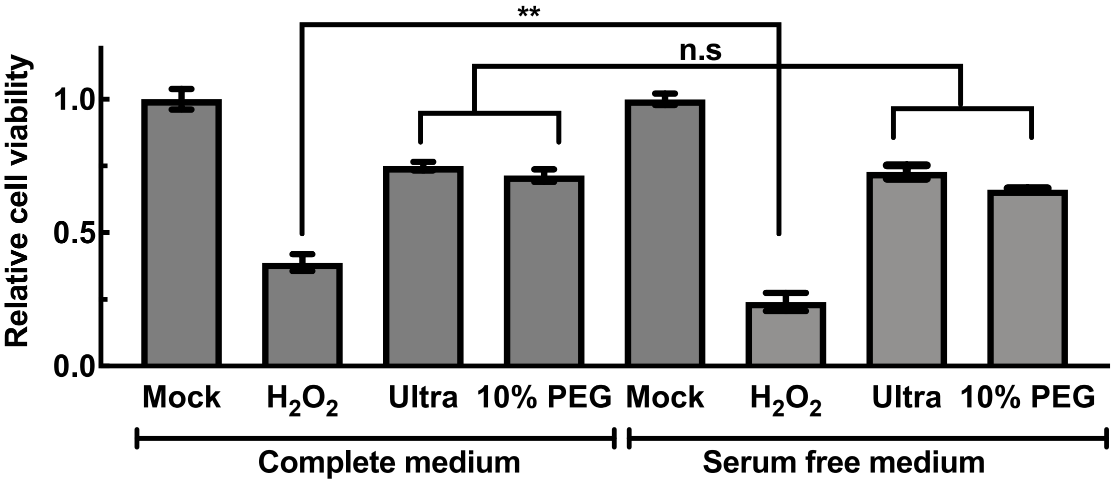

Fig S6: The antioxidant activity of ultra and PEG ENPs is not affected by the presence or absence of serum in culture medium. Cell viability of RAW macrophages treated with H_2O_2 alone or in the presence of ENPs, in medium with (complete medium) and without serum were measured by MTT assay (n=3). **P<0.01, n.s: non-significant. Under serum free conditions, RAW macrophages showed reduced cell viability compared to treatment with H_2O_2 in serum containing medium.

Table S1:

PEG concentration	Residual PEG in PEG-ENPs (in percentage)
8%	4.4 ± 0.3
10%	5.6 ± 0.2
12%	6.6 ± 0.6
15%	7.7 ± 1.1

Table S2: Acquisition Details for the ATR-FTIR spectrum

Additional Data Treatment	2
Acquisition Mode	Double Sided, Forward-Backward
Low intensity power mode with DTGS	Off
Correlation Test Mode	OFF
Delay Before Measurement	0
Stabilization Delay	5
Wanted High Frequency Limit	8000
Wanted Low Frequency Limit	0
Sample Meas. Duration in Min.	1
Background Meas. Duration in Min.	1
Sample Scans	128
Background Scans	16
Result Spectrum	Absorbance
Resolution	4
Signal Gain, Background	Automatic
Sample Rotations	32
Background Rotations	32
Sample Scans or Time	Scans
BG Scans or Time	Scans
To do list	37
Signal Gain, Sample	Automatic
Signal Gain, Sample 2nd Channel	Automatic
Signal Gain, Background 2nd Channel	Automatic
Source Setting	MIR
Scanner Velocity	7.5 KHz
Preamplifier Gain Background	Ref
Preamplifier Gain	Ref
Optical Filter Setting	Open
Measurement Channel	Sample Compartment
Low Pass Filter	Automatic
High Pass Filter	On
External Synchronisation	Off
Extended Ready Check	OFF
Detector Setting	RT-DLaTGS [Internal]
Correlation rejection mask	0
Beamsplitter Setting	KBr-Broadband
Background Measurement Channel	Sample Compartment
Aperture Setting	6 mm

Table S3:

Factor	Ultracentrifugation	PEG Method
Cost of instrument: Ultracentrifuge versus regular bench top centrifuge that can reach at least 8,000g	Expensive	5-10 times cheaper
Cost of plasticwares: Ultracentrifuge tubes versus regular centrifuge tubes	Expensive: Each SW32 ultracentrifuge tube costs 10-15 USD per tube	Each 50ml regular centrifuge tube costs <20 cents
Cost of PEG6000	Not applicable	R&D grade PEG6000 costs ~80-100USD per Kg. Approximately, 100g of PEG6000 is required to purify 2g of ENP from ginger
Scalability: Availability of rotors and tubes to reach required RCF in larger volumes	To our knowledge, BeckmanTi45 is the only rotor which can reach up to RCF_{max} 235,000g with maximal volume 94ml per tube. Beckman type 19 rotors can accommodate up to 250ml per tube but RCF_{max} is limited to 53,900g	Commercial rotors are capable of centrifugation up to ~15000g with 1 litre sample per tube. Example: Kubota AG-1K4 rotor can centrifuge 4 X 1,000ml at 15,760 x g.
Expertise required to operate the instrument	Extreme caution is required in operation, especially proper balancing down to 1% of total weight	The balancing requirement is significantly less at low speeds such as 8000g
Availability	It is usually a part of a central instrument facility in research institutes.	Readily available in any individual research laboratories.

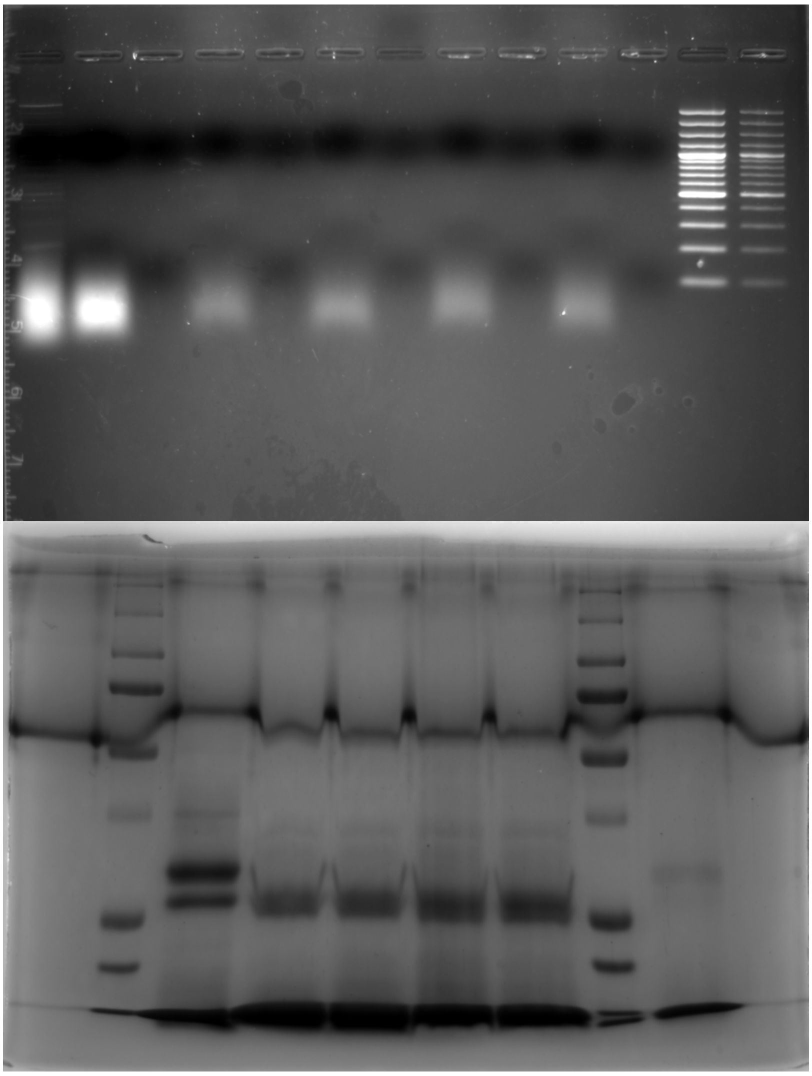

Uncropped versions of Agarose and SDS-PAGE gel images corresponding to Figure 4a and b.
